# A Qualitative Exploration of Gaps and Challenges in Knowledge and Practices of Electroconvulsive Therapy by Key Personnel in Public and Private Mental Health Units in Kenya

**DOI:** 10.3389/fpsyt.2019.00697

**Published:** 2019-10-25

**Authors:** Nabila Amin Ali, Frederick Owiti, Pius Kigamwa, Manasi Kumar

**Affiliations:** Department of Psychiatry, University of Nairobi, Nairobi, Kenya

**Keywords:** electroconvulsive therapy, knowledge, practice, guidelines, barriers

## Abstract

**Background:** Evidence-based research for electroconvulsive therapy (ECT) practice in Kenya is scarce. This has seemingly stifled knowledge with regard to ECT practice among key personnel in the country. Research shows that evidence-based guidelines not only harmonize clinical practice in a certain region but also improve health outcomes and quality of clinical decisions made by key personnel. This study aimed at assessing knowledge and administration of ECT by key personnel in psychiatric units in Kenya.

**Method:** This is a qualitative study targeting multiple stakeholders in mental health facilities. The study was undertaken in three counties: Nairobi, Nakuru and Eldoret. Snowballing sampling method was used to interview 33 targeted respondents who work in ECT departments or actively interacted with the procedure in both private and public facilities. Researcher-designed respondent profile questionnaire and interview guides focusing on knowledge, practice and barriers in delivery of ECT were used as tools. Data collected were transcribed from the audio recordings. Thematic and content analyses of these semi-structured interviews were carried out based on the patterns that were noted across the data collected. The interviews were read by the research team and re-read to highlight the core ideas. Findings were presented in form of themes, which were illustrated along with representative verbatim quotations.

**Results:** Overall, the key personnel were knowledgeable about ECT in different stages of the procedure, but we noticed methodological incongruence in their practice with regard to the pre-ECT preparation, stimulus dose calculation adequacy of seizure and in the procedure for dose adjustment of psychotropic medication before and after ECT sessions. The identified barriers to the uptake of evidence-based practice were lack of infrastructure, inadequate funding, lack of adequate training and negative perception by patients, relatives and even some participants.

**Conclusion:** Though key personnel in this study showed that they had knowledge on ECT administration, lack of standard guidelines on ECT practice led to lack of standardized training on the procedure hence the methodological incongruence. Inadequate infrastructure, knowledge and negative perception towards the procedure seemed to interfere with uptake of ECT as an intervention.

**Recommendations:** The study makes the following recommendations: adoption of a guideline by psychiatrists, intense training on ECT, specialized training for nurses in ECT and dose calculation for psychiatrists and registrars. Funding should be made available for new ECT machines. Lastly, education and awareness creation should be done about ECT to help deal with negative perception towards the intervention.

## Introduction

Electroconvulsive therapy (ECT) is an essential intervention in psychiatry (1) with a history dating from 1938 when it was introduced by Lucio Bini and Ugo Cerletti, who are respectively, an Italian psychiatrist and an Italian neurologist (2). The procedure involves application of tiny electrical currents through the scalp to induce therapeutic seizures (3). The National Institute for Health and Care Excellence (NICE) guidelines and Royal College of Psychiatrists (RCP) recommend ECT as a first-line intervention for patients with life-threatening conditions like severe major depressive disorder (MDD) with suicidality and depressive illness associated with stupor, psychosis or marked psychomotor retardation. ECT should be considered as a second- or third-line treatment in patients with catatonia or protracted or severe manic episode or in patients with treatment-resistant schizophrenia. It is also prescribed when quick recovery is a priority or in patients with prior good response to ECT (4, 5). Treatment-resistant psychiatric conditions are those that do not respond adequately to two or more treatment trials used for a period of 6 weeks or more at the maximum effective dose (6).

To administer ECT, a multidisciplinary team of experts who have knowledge and skills on ECT is needed, which includes an anesthesiologist, a psychiatrist and psychiatric nurses. Other medical practitioners such as neurologists and cardiologists, among others, should be available for consultation and are required to have adequate knowledge on ECT. A multidisciplinary team is essential in case of co-morbidities such as cardiovascular system conditions, epilepsy and less commonly, dental and oral injuries post-ECT due to clenching of the jaw; risk increases if patient already has a dental condition (6). For this reason, it is extremely important for this team to be aware of the standardized ECT guidelines and procedures, which state that clinicians should be trained on ECT and guidelines should be followed to minimize adverse effects related to ECT and maximize its therapeutic effect (7).

Clinical guidelines, on the other hand, have become part and parcel of clinical practice worldwide. Research shows that evidence-based guidelines not only harmonize clinical practice in a certain region but also improve health outcomes and quality of clinical decisions made by key personnel, consequently reducing morbidity and mortality and improving quality of life in patients with certain conditions (8).

### ECT Utilization Worldwide

ECT utilization rate has oscillated over the years since its introduction. It was at its peak between 1950s and 1960s, a time when there were few alternative medications for severe psychiatric conditions. With the introduction and evolution of neuroleptic medications and increase in stigmatization of ECT, its use drastically declined during the 1970s and 1980s worldwide. With time, it was realized that these medications were not a magic potion and that there was a need of ECT for a particular group of psychiatric patients (2). Consequently, approximately one million patients with psychiatric illness receive the treatment annually throughout the world (9). However, the intervention continues to be underutilized in some parts of the world for a number of reasons, such as its historical unpleasant experiences; divergent opinions of health care providers, patients and their relatives about ECT and also the inconsistency between ECT guidelines (10). Despite its existence for more than seven decades, between 2000 and 2003, there are countries such as Cambodia, Georgia, Lebanon and Brunei, where ECT was not available (11); and in Slovenia, ECT was banned completely (12). The ban was influenced by ALTRA, a nongovernmental organization in Slovenia, which focuses on rights of patients, claiming that ECT is a human rights violation due to its intrusive and irreversible effects on the brain, a claim that is not supported by evidence.

ECT practice demonstrates a heterogeneous presentation in its practice worldwide, with some countries utilizing it more frequently than others. In general, research has shown that ECT is provided in less than half of all psychiatric facilities within the same country; for example, in the Netherlands (9) and Hungary, 23% and 47% of all the psychiatric units provided ECT services, respectively (13). On the contrary, Spain (55%) (14), the Czech Republic (67%) and Slovakia (92%) are some of the countries where majority of the psychiatric units provide ECT services (15). Interesting to note is that in Greece, very few public hospitals (*n* = 4) provided ECT in 2007 and those were exclusively teaching hospitals; the rest were private facilities (*n* = 14) (16).

### ECT Devices and Administration

The American Psychiatric Association (APA) recommended the use of modern ECT devices, putting into consideration the increase in cognitive adverse effects caused by sine waves (4, 17). These modern ECT devices are featured with a fixed current, which delivers continuous currents, despite resistance caused by patient’s scalp. Brief (0.5–2 ms)/ultra-brief (0.2–0.5 ms) pulse widths are the ideal waveforms. Unlike the traditional sine waveform (8.33–10 ms), brief waveform has a better cognitive profile (18, 19). The rapid rise and fall of brief pulse waveform is equivalent to the action potential in the brain neurons, hence requiring less overall stimulus intensity than does a sine-wave stimulus (20). There are some devices that are featured with electroencephalography (EEG), which is important for monitoring seizure adequacy (4).

Modern ECT devices have hence been adopted and used by many countries. In Canada, for example, nearly all facilities offering ECT services use brief and ultra-brief pulse devices (21). Sine-wave devices, on the other hand, are used in countries such as Russia, where about one third of all facilities had outdated machines in 2005 (22), while in Belgium, about a decade ago, the percentage was at 30%, but this has since changed as per the study done by Sienaert et al., which showed that the usage of sine-wave devices has markedly reduced to one device (6.7%) (23). In Greece, more than half of all psychiatric facilities use devices delivering brief pulse square wave (16). In a psychiatric hospital in Egypt, Mecta spectrum 4000M was the preferred modern ECT device (24). While in Sudan and Nigeria, Ectron series 5 and 6 UK-manufactured machines and Ectron series 5a were preferred over modern ECT devices, respectively (25) (26).

In the USA, between 1940 and 1970, ECT was commonly done in an outpatient setting and sessions were not scheduled in advance but rather when needs arise and continuation ECT was the order of the day (27). To date, ECT is still considered as an outpatient procedure despite the hospital admission a day before or later after the intervention as per the Ontario convention, especially if the patient needs continuation or maintenance ECT sessions. As for the acute and very unstable psychiatric cases, ECT is done as an inpatient procedure. In Canada, for example, about 90% of all facilities performing ECT did it on both outpatient and inpatient bases, while the remaining 10% administered it as an inpatient procedure (28).

The scope of this research is not intended to determine efficacy or adverse effects of ECT but aimed at understanding ECT practice in Kenya with regard to the entire ECT care. The researchers explored the differences in knowledge and in administration of ECT by key personnel in public and private facilities and determined whether this practice conforms to ECT guidelines used in Kenya. The guideline to be used is mapped on the RCP ECT guideline; currently, Kenya does not have a guideline of its own on the use and monitoring of ECT procedure. The researchers also explored barriers to adherence to the guideline.

## Methods

### Study Design and Methodological Approach

This study was a health service research and qualitative in design, which was approved by Kenyatta National Hospital and University of Nairobi Ethics Review Committee on 2nd October, 2017 (Approval No. P331/06/2017). The data were collected by the first author who worked on this paper as her Masters in Medicine thesis project. The study protocol was developed by MK, FO and PK, who acted as her mentors. MK focused on mental health services perspective, while FO and PK are psychiatrists who were interested in knowledge and practices associated with ECT use. The researchers used a respondent profile questionnaire and individual stakeholder-specific interview guides to gather information from clinical staff affiliated with ECT department in both public and private practice in Kenya. The researcher preferred this data collection method, as it helped in facilitating the collection of essential information from ECT experts regarding ECT practice, given that there is scarce information about the characteristics of ECT practice in Kenya. The study was conducted in three counties in which ECT is currently being provided in Kenya, namely, Nairobi, Nakuru and Eldoret. These three counties, out of the 47 counties in Kenya, are the only counties where ECT service is offered. This information was provided by the Kenya Ministry of Health. A total of nine psychiatric units provide ECT services in the three counties, the majority of which are in Nairobi, the capital city of Kenya. The research area of this study included all public and private hospitals providing ECT.

### Participants, Setting and Interview Type

Snowballing method was used to recruit the targeted respondents. Participants who met the criteria were taken through participant’s information, which explained the purpose of the study and a written informed consent was taken from the willing key personnel. The researcher included key ECT personnel (psychiatrists, psychiatric registrars, psychiatric nurses and anesthetists) from public and private facilities offering ECT in the country. As a qualitative study, the sample size was not expected to reflect a sample of the actual population to tease out attitudes, knowledge and practices associated with ECT in Kenya. The researcher interviewed a total of 18 consultant/resident psychiatrists, 11 nurses and four anesthetists. Table 1 summarizes respondents’ profile. Once the participants were identified, a face-to-face interview was conducted on the agreed date after signing an informed consent. At times, follow-up interviews were carried out if some stakeholder ideas needed further exploration.

**Table 1:** Summary of respondent profile.

Respondents Profile Males = 17 Females = 16			
Years in practice	Frequency	Cadre	Frequency
*1–4 years*	*13*	*Consultant psychiatrists*	*10*
*5–9 years*	*5*	*Resident psychiatrists*	*8*
*10–14 years*	*5*	*Psychiatric nurses*	*11*
*15–19 years*	*5*	*Anesthetists*	*4*
*20–24 years*	*2*		
*Above 25 years*	*3*		

### Data Collection Procedure

The data collection process took 12 weeks, from 15th February, 2018 to 30th April, 2018. The first 4 weeks were dedicated to respondents within Nairobi, while Weeks 5 to 8 were put aside for respondents out of Nairobi. The last 4 weeks were reserved for key informants who at the time of the study were out of the country or on leave or for any eventualities that may cause hitches in the data collection process.

### Data Collection Instruments

#### Respondent Profile Questionnaire

A researcher-designed respondent profile questionnaire was used to give a description of the respondents. The questionnaire ascertained if these key personnel were affiliated with ECT department, the period they have worked at the ECT department, under what capacity, where they got their skill and knowledge on ECT, including their gender. Refer to **Supplementary Appendix 1**.

#### Researcher-Developed ECT Interview Guides

In-depth interview guides were used to guide the discussion on ECT practice in Kenya. The participants were grouped into three, each group targeting a specific cadre of clinical staff. Interview guides with open-ended questions ranging from 1 to 12 with subsections were used for each category of key informant. See **Supplementary Appendix 2**.

### Quality Assurance Procedures/Pretest

A pretest was carried out where a respondent from each cadre of clinical practice was interviewed and their responses were assessed. Their understanding of the questions being asked was also assessed by the researcher to ensure that they fully understood the questions and if not, then adjustments were done in the wording of the questions for more accuracy. Data collected using audio recordings were translated and transcribed and thereafter entered into a password-protected Microsoft Access Database. Once entry was complete and hard copies of interviews were printed out, data on the hard copy and audio recordings were compared with the entered data to ensure accuracy. Content and thematic qualitative analyses were carried out based on the patterns that were noted across the data collected. According to Nowell et al. (29), thematic analysis is a useful method for examining the perspectives of different research participants. When conducting such a research, the researchers’ sensitivity and immersion in the study along with their ability to become an instrument of analysis, making judgments about coding, theming, decontextualizing and recontextualizing the data are a critical part of the work (29). Findings were presented in form of themes, which are illustrated through verbatim quotations.

## Results

### Respondents’ Profile

Out of the 40 participants who were approached, 33 participants were recruited in the study. They were as follows: 10 were consultant psychiatrists, 8 resident psychiatrists, 11 nurses and 4 anesthetists. All the 10 psychiatrists have part-time private practice; one of the anesthetists and four nurses were full-time employees in different private facilities. Participants were interviewed at different locations depending on their comfort, but most were at their work stations. There were more male participants than female: 17 and 16, respectively. Most of the nurses who participated in the study were female. There was an equal distribution of male to female ratio when it came to psychiatrists/registrars, a ratio that is not reflective of the consultants working in both private and public institutions; there are slightly more female than male psychiatrists in Kenya at a percentage of 52.4% and 47.6%, respectively. The same ratio of female to male was found with anesthetists who participated. Among the doctors, there were more consultant psychiatrists than registrars who participated in the study. Neurologists did not meet the study criteria and therefore, were not included in the study. Most of the participants indicated that they had ≥5 years’ experience in administering ECT. With regard to their professional qualifications, all the nurses interviewed indicated that they were qualified ECT nurses. However, it was later noted that most of them had not been specifically trained on ECT but had gained experience working in the department or attended a continuing medical education (CME) on ECT. The same was found with the anesthetists. See **Table 2** for the key domains under which interview probes were carried out.

**Table 2:** Interview domains probed with each stakeholder.

Psychiatrists	Asked about which patients were typically given ECT, the type of ECT machine/waves used, how to determine stimulus dosage to use per patient, adjustment of medication before and after ECT, the type of electrode placement used, how to define adequate seizure and what procedure is followed in the event there is inadequate seizure, methods used to monitor adequacy of seizure and finally the availability of ECT guideline in the country.
Nurses	Participants were asked to describe the Pre-ECT preparation procedure, informed consent procedure, their role in the administration of ECT and Post-ECT procedure
Anesthetists	Were asked to explain what entails anesthetic evaluation before administration of ECT, the equipment available in the ECT procedure rooms, the type of medication used before, during and after ECT, what parameters are monitored before, during and after ECT and the availability of a guideline on anesthetic procedure.

It was noted that most key personnel did part-time private practice in different facilities and hence, had experience in ECT care from two or more psychiatric units providing ECT services the reason being that there are few psychiatrists to serve the high need for mental health services in the country, hence forcing the few psychiatrists to offer their services in more than one facility.

#### Overall Emergent Themes on Knowledge and Practices of Key Personnel on ECT in Public and Private Facilities in Kenya

##### Cognizance of Overall Theory and Methodology

The knowledge of the participants was assessed using semi-structured interview that was mapped on key ECT features, areas that need scrutinizing based on Kenyan medical and psychiatric contexts. We had inputs from all three co-authors, two of whom are leading ECT practitioners in Kenya, who are exposed to public and private psychiatric services. All iterations were based on qualitative expert opinion feedback. We will follow up by using a structured quantitative survey based on this formative study.

Participants’ response to our questions on specific areas of the ECT procedure showed that, by and large, the key personnel in both private and public facilities demonstrated reasonably good awareness and knowledge in the procedure at different levels and stages of the ECT procedure. This could be attributed to training, extensive reading on ECT, familiarity or long-term practice in providing ECT or assisting in providing ECT in their different areas of expertise. The nurses in their interviews demonstrated that they were indeed knowledgeable on the key aspects of pre-ECT preparation. The anesthetists understood the importance of using the correct anesthesia and of noting what type of medication the patient was on, which could actually affect the efficacy of ECT. The same sense of awareness was also noted in post-ECT care, particularly with regard to patients who are put in recovery position with vital signs being monitored. This includes checking up for any adverse side effects, eventually managing the patients in the ward as per their doctor’s prescription.

Doctors were also found to be knowledgeable on the general administration of ECT, indications, contraindications, what type of baseline investigation to order and the type of electrode placement method used in their facilities. All of them except one participant had confidence that ECT is effective and that it has a predictable outcome especially in patients with MDD and catatonia. Most of our participants shared that the proof of the acceptability and effectiveness of ECT was in the higher rates of success as opposed to failure after ECT (something we took at face value, as we did not look for records or demonstrated evidence). **Table 3** highlights the sub-themes together with verbatim quotations from the respondents under the emergent theme on knowledge of ECT practitioners.

**Table 3:** Emergent themes and verbatim quotations under Knowledge.

A. Themes under Knowledge	Verbatim Quotes
*Cognizance of overall theory and methodology”* *Among Psychiatrists & Registrars*	✓ *ECT Indications & Contraindication Knowledge* “From my knowledge I think very suicidal patients, depressive patients that are very suicidal, patients who are very unstable when they are pregnant, schizophrenia that is not responding to drugs, patients who are not able to take oral medication, postpartum psychosis it’s actually an indication…” *Registrar Psychiatrist* “you cannot do ECT…I guess in conditions like multiple oesteosclerosis or any conditions where there are any fractures of the bones especially on the long bones…aah…the other contraindications are the…many have something to do with issues that are more to do with anesthetist where the patient that they want to do, is allergic to the anesthesia because it is done under general anesthesia in our session…aah…what else can be a contraindication? Contraindications are procedural…but generally speaking, I don’t find that you have a question on this...” *Qualified Psychiatrist* ✓ *Effectiveness of ECT* “ECT works…it is a very effective treatment more so for depression and bipolar especially in mania. It is a very good intervention and I have also found it very helpful in mothers, postnatal mothers and effective in the treatment of persistent schizophrenia…in the books it says it is not effective and I was also resistant initially but in my experience I have found that it helps…treatment resistance schizophrenia”*Qualified Psychiatrist* ✓ *Importance & Reasons for Preference of Bilateral Electrode ECT Machines* “It’s bilateral and not unilateral, it has been deemed to be more effective than unilateral and therefore in our setting I have never seen unilateral it’s always been bilateral”…*Registrar Psychiatrist* “Well they say that unilaterally is not as effective as bilateral… I think that’s the main issue. But I have never seen unilateral being used on a patient and even when we found them using the direct method, it was still bilateral…but I assume that may be its more effective than the unilateral”*Qualified Psychiatrist*

### Methodological Incongruence

There were some variations in practice between public and private institutions; for example, in public facilities, ECT is mainly done by the psychiatric registrars without any supervision, while in private hospitals, consultant psychiatrists administer the intervention. ECT is mainly done as an inpatient procedure in public hospitals, while in private hospitals, the intervention is offered to both inpatients and outpatients. Some differences also emerged in certain aspects of pre-ECT workup and also during ECT administration. With regard to pre-ECT workup, for example, in some institutions, there were no investigations done prior to an ECT administration. Another difference that was noted under pre-ECT preparation was the shaving of the head procedure. Notably, all private institutions did not shave their patients’ heads as a prerequisite for ECT administration, while most of the public institutions did this as a routine for those patients who had long hair and were prescribed for ECT. In these cases, they would either shave the hair around the temporal region or the whole head to enable proper placement of electrodes at the temporal region. Failure to comply meant no ECT for that patient on that particular day till he/she gives consent for the same. Other differences that were noted were in the different methods adopted for stimulus dose calculation. Though some doctors and registrars in both public and private facilities considered the half-age-rule method, most of them just administered a similar dose for every patient. It is however, important to note that the half-age-rule stimulus dosing strategies are basically dependent upon the model of the machine used (30). But generally speaking, there was lack of knowledge on the type of machine used and the type of wave produced.

Incongruence in practice that was also noted during ECT administration was mainly with regard to how doctors managed the lack of or inadequate seizure. It was appreciated that most of the doctors had successful attempts most of the time they administered ECT. However, for those who were initially not successful, they repeated the ECT administration either at a day later or immediately up to a maximum of three attempts. **Table 4** highlights the sub-themes together with verbatim quotations from the respondents under the emergent theme on practice of ECT.

**Table 4:** Emergent themes and verbatim quotations under practice.

Themes under practice	Verbatim quotations
*Methodological Incongruence*	✓ *During Pre ECT Work up - some institutions did some investigations before ECT while other didn’t.* “we don’t do much; unless we query and if we find there is anything, then we can do a CT scan and see if there is a problem…(inaudible)…yes, but if there is a query then we will do the investigations otherwise we assess the patient, book for ECT and administer…*Nurse* ✓ *No consistent method of Dosage Calculations* “Yes, we calculate the dose with The age of the patient. since we’re doing bilateral it is always half the age of the patient so if the patient is 20 years the initial ECT will be half that is 10...And then for the subsequent ECTs the doze will be half of the previous so that’s how we calculate”…*Psychiatrist* “So you use the pre-selected formula based method…yes”…*Psychiatrist* ✓ *Action to take if adequate seizure wasn’t achieved - some psychiatrists increased the dose and repeated ECT while others waited another day* “You just write failed and then you increase by 50% on the consecutive session... i have never repeated it the same day on the same patient that was what we were taught.”…*Resident Psychiatrist* “First, make sure your connections are okay, the machines are in order and the electrodes are properly placed. If those ones are in order then you can usually repeat” …*Resident Psychiatrist* ✓ *Others stopped the neuroleptic medication before ECT; others just reduced the dose…* “They will just continue with the medication, we may lower the medication because of ECT; even if there are mood stabilizer, ok, they might lower the seizure threshold but we rarely stop the medication when we are doing the ECT…we rarely do it, because we still get the seizure and so we rarely adjust”…*consultant psychiatrist* “I will be very accurate to see that its 12 hrs before the ECT…that’s what they do on the ground…because if I write 8am in the morning, they will still give the patient the morning dose and only remember the evening dose and which they will not give and so 12 hrs before the ECT is when the medication will be stopped… resident psychiatrist” ? *Shaving of the head..* “There are patients that we shave but we don’t shave the patients all the time…if you get that the patient has a lot of hair then we will shave him/her at the temporal lobe” …*Psychiatric nurse* “Yes, normally tells them early enough, only we need this part of the hair and those who are not willing to cut off their hair, so some of them will end up missing ECTs because they will create a big issue out of it there is nothing much we can do”….*Psychiatric nurse*

Some similarities between private and public institutions were also found; for instance, in both institutions, general anesthesia and muscle relaxants were used prior to ECT administration and no facility had a backup ECT machine in place in case the machine in use breaks down. It was also noted that in both facilities, ECT was done on alternate days except weekends for public institutions. In addition, a course of ECT in both institutions would mean six ECT sessions, unless improvement is achieved before the completion of the sessions or if the patients/relatives are unable to pay for all the six sessions.

### Adoption of ECT Practice in Kenya in Relation to the RCP ECT Guidelines

With regard to adoption of ECT guidelines and implementation in public and private hospitals in the country, we found that there was no specific guideline or policy structure around its stipulated practice in any of the facilities providing ECT. The hospitals had not adopted the RCP ECT guidelines and some of the providers were not fully aware of this specific one or other guidelines for that matter. All the clinicians who were interviewed indicated that they had no documented guidelines that they used while providing ECT. Many of the key personnel get their knowledge from psychiatric books or reading online. Refer to **Table 5** for the vignettes that support this theme.

One of our policy participants shared that the Ministry of Health has appointed a task force to work on Kenyan mental health policy guideline, which will include a policy and practice regulation chapter on ECT. Thinking through of the policy may easily take approximately 2 years or so.

**Table 5:** Emergent themes and verbatim quotations under Guidelines.

Theme under Guidelines	Verbatim Quotes
*“Lack of a specific guideline or policy structure such as SOP around its stipulated practice”*	“We need guidelines so that at least new, young psychiatrists they know where to start…*Psychiatrist* A guideline for ECT? Not that I know of including all interventions where… *Psychiatrist* “ECT guidelines…where have I seen guidelines…I think I have seen the requirements”…*Psychiatrist* “We will be dealing with standards and clinical guideline I think even a task force has been appointed to work on the clinical guidelines so initially we will not do a standalone clinical guideline on ECT we look for everything and we may have a chapter on ECT”…*Psychiatrist*

#### Barriers to Adherence to the Evidence-Based Practice of ECT

Besides the lack of set guidelines, the study also looked at other factors that hindered adherence to evidence-based practice of ECT and one of the barriers that we could identify was *inadequate resources*, which could potentially come in the way of developing ECT as a standard well-organized procedure. This was noted particularly with regard to equipment and infrastructure in the public hospitals and also in some of the private hospitals. Lack of availability of nursing and specialist staff was noted in both private and public institutions. It was also noted that most of the patients who needed ECT had to incur very high costs and hence, it was deemed difficult to put patients on maintenance ECT due to lack of adequate resources to constantly administer the treatment. For instance, it was noted that in one of the public facilities, there was no recovery room and that patients were taken from the theatre and pushed into the corridor.

Another emergent theme was “lack of adequate training” around this procedure at all levels of hierarchy. As indicated earlier, most of the participants were not formally trained, but instead, they had learned through observation and practice. Finally, *stigma and negative perception towards ECT* was emergent from the study. **Table 6** summarizes the themes under barriers to adherence of evidence-based practice of ECT.

**Table 6:** Emergent themes and verbatim quotations under Barriers.

Theme under Barriers	Verbatim Quotes
“*Inadequate Resources.” That has lead to poor infrastructure, poor planning and delay in services and in some cases lack of essential supplies*	“Some of the challenges are for one space…space to provide ECT is a problem because the room is small so it can only accommodate a small number of personnel inside, second; lack of qualified personnel. With regards to personnel, we need to have personnel with psychiatric background, supplies are inadequate and any support from the administration is good and may be what we are looking for is a new ECT machine”…*Psychiatrist* “The main challenge of ECT is actually the cost of ECT machines; the cost for …like in this hospital, we have asked them to give us an ECT machine for years and they have never given us. So the availability of ECT machines and cost cutting are the biggest” *… Psychiatrist* “We face some challenges for example nowadays we have many patients booked for ECT but we only have one machine so patients tend to wait for some time and if the machine breaks down the patients have to wait for the machine to be repaired; there is no back up. …*Psychiatric Nurse* “And in the wards, nothing much and pharmacy sometimes we lack some drugs.”…*Anesthetist*
“*Lack of adequate Training.*”	Interviewer: “Okay, while I was interviewing the registrars, they told me some of you… you have taught them how to do ECT… Respondent: This is about the settings of the machines and how to hold the electrodes but administering……….No, I normally instruct them on how to press, where to press as I have seen it being done before”…*Anesthetist* “The only training is observations…you come there and observe…and then you read on your own about ECT”…*Resident psychiatrist.* “Basically the knowledge we have is good but with time you tend to forget that’s why there is a need for continuous medical education. You may assume you know but you don’t know.”…*psychiatric nurse*
*Stigma and Negative Perception by relatives, patients and even by medical staff*	“Sometimes you may want to give ECT but the owner of the patient refuses because of what they have heard”...*psychiatrist* “The ECT practice is a stigmatized intervention it requires a lot of public education, I have seen ECT recently on BBC on TV showing the whole procedure although it has been used by the anti-psychiatrists that we are shocking patients.”…*male psychiatrist”*

## Discussion

Our study highlighted a number of knowledge and practice-related challenges in facilities that offered ECT in Kenya. Less than half of all psychiatric units provide ECT in the country and out of the 19 psychiatric units we have in public facilities in Kenya, only four (23.5%) provide ECT services. From the four facilities, two are teaching facilities, one is a military hospital and one is a county hospital. All five private institutions providing the service in Kenya are in Nairobi County. Similar results were found in a study carried out in Greece where very few public hospitals provided ECT and these were mainly teaching hospitals while the rest were private facilities (16). In contrast to Greece and Kenya, Australia (66%) and Denmark (100%), both of which are highly developed countries, more than half of the facilities providing psychiatric services offered ECT (9). A comparable study done in South Africa found that in majority of the institutions included in the study, ECT was administered by consultant psychiatrists, but in some cases, general practitioners and resident psychiatrists would administer the intervention (31). In contrast, another study found out that anesthetists or nurses would administer the intervention in some facilities. This finding is similar to that of the study conducted in Sudan where a trained senior nurse is allowed to administer ECT (32). Given the paucity of trained psychiatrists, this could be a way of promoting task-sharing.

### Knowledge of Key Personnel on ECT

The study revealed that there was cognizance with regard to knowledge particularly among the psychiatrists who participated in the study. Generally, the professionals interviewed indicated that through their vast experience, frequent prescription of ECT and also use of ECT machines in the hospitals or in their private practice and more so the positive outcomes from patients, they indeed had overall knowledge about ECT. Nearly all the psychiatrists and registrars seem to have extensive knowledge on the indications and contraindications of ECT. Similar results have been revealed from other studies. A study carried out to determine the knowledge of and attitudes towards ECT among psychiatrists and family physicians in Saudi Arabia reported that, overall, psychiatrists had better understanding and knowledge about ECT unlike the general physicians. They also found knowledge gaps in regard to the seizure adequacy (32). Another area of knowledge that was assessed was in understanding the level of efficacy that ECT had over the use of neuroleptics or antipsychotic medications and similar to the Saudi Arabia study, the psychiatrists in this current study fully understood and exuded confidence in the ability of ECT to relieve symptoms quicker and improve patients’ conditions faster than medications-only treatment. Both studies also showed that the psychiatrists and in this current study, all the participants knew that ECT could only be administered after the patient was under general anesthesia. There was general consensus from both studies that some conditions like heart-related disorders were contraindicated in ECT and that frequent sessions of ECT were ideal for better results. As for whether the seizure administered was adequate, the key personnel understood that just observing a seizure is enough to achieve a therapeutic effect of ECT no matter the strength of the seizure. Some even thought that the stronger the seizure, the better the outcome. However, this is a controversial matter. Similar to those in the study in Saudi Arabia, the psychiatrist and registrars indicated that they would give six and rarely eight sessions to a patient averaging to two or three per week.

Anesthetists also seem to be knowledgeable with regard to the anesthesia that should be administered before ECT is done and general importance of their presence during the ECT administration. A study done to determine the importance of improving the anesthetic depth assessment during ECT with bi-spectral index monitoring further showed the importance of use of proper and effective anesthesia during ECT (33). Another study done in India that sought to review available literature on anesthesia use and its importance pointed out that it improved the clinical efficacy of ECT and patient’s ability to tolerate the seizure and electrophysiological variables/parameters (34). The same sentiments were shared by the anesthetists in this current study.

This study also revealed that nurses had mostly pre-ECT and post-ECT care knowledge than knowledge on the ECT technique itself. They were mostly directed on what to do by the psychiatrists and the registrars. A study done in London to assess nurses’ knowledge of and attitude to ECT found a significant correlation between years of experience and exposure in the ECT department, with more knowledge and positive attitude towards the intervention (35). This was similar to the findings in this current study. A Nigerian study indicated that nurses generally seem to have a lower grasp on knowledge regarding ECT (36). Unlike the participants in this study, they were well informed about their role in pre-ECT preparation and post-ECT care. The Nigerian study also revealed that the nurses did understand the principles of ECT, but they also believed that it was outdated and cruel and could possibly cause brain damage, in contrast most of the nurse participants in this study who believed that it was efficacious and reported such negative sentiments stemming from the relatives’ point of view.

### Practice Gaps in ECT Administration

The study revealed that there was methodological incongruence or gaps when it came to ECT practice. Generally, pre-ECT workup involved a number of investigations and tests being ordered by most psychiatrists and registrars; however, not all the practitioners ordered the tests. As per the RCP ECT guidelines, these are vital tests that need to be available for affirmation of the patient ability to undergo ECT. A misguided pre-ECT workup procedure that was practiced by some key personnel in this current study was the shaving of the hair. As indicated in the ECT guidelines by the British Columbia Ministry of Health, it is noted that shaving the head is not a prerequisite for ECT to be administered (37). However, this current study revealed that in some cases, in public facilities, patients’ refusal to be shaved was met with delay in procedure until the patients reconsidered; otherwise, no ECT is administered. Based on the fact that bilateral placement of electrodes does not in any way get interfered with by patients hair, this procedure needs reviewing in most mental health facilities, as it delays adequate and appropriate management of the patient, hence increasing their hospital stay. Additionally, this is a violation of patients’ rights, as there is no basis for such practice.

Fundamental concerns were also articulated around ECT machinery-related knowledge. The participants did not have knowledge about the kind of machine that they used to administer ECT. The implication of this gap was directly related to administration, because they were also not aware of the nature or kind of electrical wave form produced by the machine and that would then interfere with the stimulus dosage calculation. Few key personnel were aware that newer ECT machines produced brief/ultra-brief pulses as opposed to the older ones that produced sine waves. Liaison with medical instrument or supply companies could help in ensuring adequate training on ECT machines.

It was noted that some participants calculate the stimulus dose using the patients age or half age (38), which, according to a study carried out in China on psychiatric patients, is basically associated with overestimation of seizure threshold in most cases and hence associated with more cognitive side effects (39). This could probably explain the frequent memory distortions and confusion that occurred after ECT. Even more concerning was the fact that other practitioners were using a similar stimulus dose (fixed dose method) for all the patients they had prescribed ECT, a practice that should be discouraged. The latter was a practice noted among most practitioners in this study. Very few practitioners were actually using the empirical titration method to calculate stimulus dose. This method is associated with low seizure threshold determination; hence, patients might end up needing subsequent administration of electricity current as seizure threshold is determined. However, it is associated with better results and less cognitive side effects (39).

### Adoption of ECT Practices as Per ECT Guidelines

One of the most noteworthy findings in this study was that there are no standard guidelines for ECT practice in place in the hospitals providing ECT services. None of the participants indicated that they were aware of a pre-existing guideline in Kenya that was adhered to while administering ECT. Therefore, though the practice was partly right and efficacious in most cases, a lot of procedural processes were not followed. Similar gaps have been reported in other countries but to a smaller extent. In a study that was undertaken in Canada, it was found that 16% of the institutions that participated in the study were not following any standard guidelines during ECT (40). Less than 30% of the participants of this study indicated having some written policy for managing concurrent medications during ECT and practice was quite variable regarding individual psychotropics. Lack of guidelines leads to increasing methodological incongruence in both private and public institutions, as each stakeholder or institution develops its own style and procedure without necessarily looking at its evidence-based practice.

### Barriers to Adherence to the Evidence-Based Practice of ECT

The other main barrier to evidence-based practice in ECT was lack of adequate training of personnel in carrying out ECT. The study revealed that knowledge from inception of ECT administration in Kenya has mainly been passed from colleague to colleague (medical officers, senior registrars, consultants, and anesthetists) and from reading basic books. This finding has been noticed in other context too, in a study carried out to explore ECT from both sides, looking at late to early careers in psychiatry; it was reported that there was a severe lack of training in ECT from the medical school levels (41). This study was done in the USA. In this current study, a number of participants had just attended a number of CMEs from which they had basic but not clinical training in ECT. Another related study that looked at the use of ECT in Central and Eastern European countries also reported lack of clinical training in ECT as a problem in the region (15). A practice like shaving of hair is an implication that lack of continuous training seems to have led to key personnel’s fixation on learned methods that do not necessarily add value to the patient’s well-being while undergoing ECT. This pre-ECT workup, which is deemed unnecessary, can be a major hindrance to most female patients and can ultimately lead to reasons for not doing ECT (42).

Cost associated with the ECT procedure was another barrier found in this study. It is expected that a patient undergoes two to three ECT sessions a week; however, it becomes very costly for the caregivers who have to cover the cost of treatment. A session of ECT procedure costs about 20$ in public facilities to between $180 and $300 in private facilities. Considering that 38% of Kenya households earn less than $100 and 27% earn between $100 and $250 per month (43), the above cost of ECT is way above their purchasing power, making these privatized arrangements unaffordable. Therefore, most of the participants mentioned that they had to sometimes change to medication-only treatment due to their inability to cover cost. This also made most of the psychiatrists and registrars consider ECT as a second-line treatment for most indicated disorders unless in psychiatric disorders that absolutely required it as a first-line treatment, for example, depression in pregnancy and puerperal psychosis.

Inadequate infrastructure was also a hindrance to evidence-based practice of ECT, a concern shared by most countries in Eastern and Central Europe (15). Most institutions in this study did not own ECT machines and hence, patients were referred to other facilities that had the machine. Most of the private facilities charge highly for the treatment. Public hospitals reported high numbers of patients who are prescribed for ECT, but due to availability of only one machine, the delay in treatment delivery was phenomenal. Some public facilities had broken-down machines that needed repairs, but due to unprecedented future costs and local unavailability of spare parts, delay in repairs was experienced. This resulted in ECT administration for prescribed patients to be constantly postponed.

Though basically a patient factor, stigma and misconception about ECT influenced by social media were also mentioned as barriers for adoption of evidence-based practice in ECT administration. Patients and their caregivers’ negative perceptions towards ECT reportedly limited the use of ECT. Similar findings have been reported; in a study that focused on Central and European countries, it was reported that ECT has been banned in Slovenia due to stigma and negative perception (15). Another barrier highlighted in this current study about the poor uptake of ECT was the fear of anesthesia by the patients.

## Conclusions and Further Recommendations

Though the key personnel in this study manifested sound knowledge in ECT administration starting from indications to contraindications, the findings clearly show a real need for a nationwide guideline and practice framework for ECT. Lack of a standard guideline in ECT practice seemed to have led to lack of a standard training on the procedure and hence the methodological incongruence. Lack of adequate or specialized training on ECT was also a major concern among the key personnel. Continued medical training on ECT also seemed to be rarely offered and hence, issues like questionable or old methods for stimulus dose calculation were noted. There was also lack of knowledge of the type of waves the machine produced and the same was found in relation to method of stimulus dose calculation. Inadequate infrastructure associated with lack of availability of adequate number of ECT machines, lack of an ideal ECT department, and subsequently costs of treatment for the patients seemed to hinder the uptake of ECT. Finally, negative perception towards the procedure also seemed to interfere with uptake of ECT as an intervention and mental health promotion and literacy was needed in this regard.

The study, therefore, recommends that Kenyan psychiatrists should adopt a particular guideline for ECT practice and partner with stakeholders like the Kenya Psychiatric Association (KPA) and the Ministry of Health in developing a local guideline for ECT. This will allow for standardization of the practice across all medical institutions. Intense training on ECT should be started from the undergraduate level and this should be based on the guidelines and evidence-based research on the procedure. This will help in ensuring that training is up to date, as medicine keeps changing. Specialized training should be offered to key personnel who work in mental health institutions (medical officers, nurses, anesthetists, etc.). More ECT machines should be acquired, particularly in government hospitals that have none or those that have outdated machines. To curb negative perception towards ECT, more awareness needs to be created on its benefits and safety. Proper psycho-education on ECT for the caregivers of the patients should also be done prior to consent signing.

## Strength and Limitations of the Study

With this study being qualitative in design, the researcher was able to get in-depth information on ECT practice from various practitioners working with ECT in Kenya. However, there was no audit of the actual practices; hence, the study does not give a comprehensive picture on ECT practice in Kenya. We have also not engaged with clients or caregivers of those who received ECT, as we first wanted to capture the proficiency of specialists who provide these services. This piece would have enriched the findings of this study further. We therefore, recommend a more comprehensive study on the uptake, efficacy and side effect profiles of ECT to include key personnel, patients and caregivers to be carried out in the future. A more structured survey on the knowledge, attitudes and practices might further highlight specific barriers and challenges. Despite these gaps, we feel that our work captures an important area needing further attention.

## Ethics Statement

The study was approved by IRB of Kenyatta National Hospital/University of Nairobi with written informed consent from all participants. Informed consent was given in accordance with the Declaration of Helsinki.

## Author Contributions

The study protocol was developed by NA as part of her MMed dissertation. MK, FO, PK read and made edits as supervisors on her study. NA did data collection. MK and NA analyzed the data. PK, FO read the themes and gave feedback. All authors read and approved the manuscript.

## Funding

This research was fully funded by the first author.

## Conflict of Interest

The authors declare that the research was conducted in the absence of any commercial or financial relationships that could be construed as a potential conflict of interest.
