# Supplementary material for: A Qualitative Exploration of Gaps and Challenges in Knowledge and Practices of Electroconvulsive Therapy by Key Personnel in Public and Private Mental Health Units in Kenya
Source: Front Psychiatry. 2019 Oct 25;10:697. doi: 10.3389/fpsyt.2019.00697 (PMC6824151; doi:10.3389/fpsyt.2019.00697)
Supplement: Supplementary file 1 [file DataSheet_1.docx]

## Appendix I: Socio-demographic Questionnaire

Study Title:

**A qualitative exploration of gaps and challenges in knowledge and practices of electroconvulsive therapy by key personnel in public and private mental health units in Kenya**

STUDY NO: ……………………… DATE: ……………………………

INSTRUCTIONS:

Kindly fill by putting a tick in the boxes provided to the questions below honestly and rest assured that no one will judge you based on your responses. In case you are not comfortable to answer a question, please indicate so and give reasons if you wish. Once the filled questionnaire is received, the researcher will go through the questions to make sure you didn’t mistakenly skip questions without indicating that you were not comfortable. Please feel free to ask if you need any of the questions explained.

The questionnaire should take about **five (5) minutes** to complete.

1. Sex Male: Female:
2. Designation
3. Psychiatric Registrar:
4. Qualified Psychiatrist:
5. Psychiatric nurse:
6. Anesthesiologist:
7. General Nurse:
8. Other (specify): ……………………………………………………………………..…
9. Are you affiliated with the ECT department/unit

YES NO

Other (Explain)……………………………………………………………………………

1. If YES in number 3.
   1. Specify the duration you have been working in the ECT Department

………………………………………………………………………………………………

- 1. What is your role?

………………………………………………………………….……………………………………………………………………..…………………………………………………………….….

1. Have you been trained on ECT? YES NO
2. If YES kindly elaborate what kind of training it was; did you take a specific course on ECT or did you attend a CME on ECT?

………………………………………………………………………………………………………………………………………………………………………………………………

### APPENDIX II: Researcher Designed Interview Guide on ECT

### SECTION 1: ECT Provider

1. Kindly tell me what your experience is with ECT from the time you started administering it?
   1. Is it usually done as an in/out patient?
   2. Please elaborate on the action mechanism and efficacy.
2. What kind of patients needs ECT?
   1. Are there contra-indications for ECT?
   2. In what conditions can it be used as a 1^st^ line intervention?
3. What type of ECT machine is used in your facility?
   1. Is it the old type or modern?
   2. Please tell me about the advantages of using brief/ultra brief instead of the old sine waves?
4. What is the practice with regards to electrode placement in your facility?
   1. Talk about other types of electrode placement positions
   2. Kindly elaborate on where exactly the electrodes are supposed to be placed
   3. What are the advantages and disadvantages of different types of electrode placement?
5. What are the key factors which determines how much stimulus should be given
   1. Do you calculate the dose needed for a particular patient?
   2. Elaborate on the dosing strategies used
6. How would you define an adequate seizure?
   1. What are the methods used in monitoring adequacy of seizures?
   2. If the electrical stimulus doesn’t result to any seizure activity or seizure is inadequate, what procedure is usually followed at your facility?
7. What guides you in knowing how many ECT sessions to give a particular patient?
8. Tell me about maintenance/continuation ECT?
9. What happens when a patient is on Neuroleptic medication / mood stabilizers and ECT is prescribed?
10. What happens in the event ECT device breaks down
11. What if there is a Power outage?
12. Kindly tell me more about ECT guidelines used in your facility?

**SECTION 2: Anesthetist / General anesthesia provider**

1. Please take me through the evaluation / preparation of a patient from the time the doctor prescribes ECT.
2. What are the medications given to the patient prior to ECT administration?
3. Give an explanation on each drug given (type, dosage, when it’s given):
4. What equipments are available in the ECT procedure room / waiting / recovery room?
5. Describe the parameters monitored before, during and after ECT procedure.
6. Is hyperventilation part of the procedure? Please explain your answer.
7. What guides you in determining the right timing for ECT administration?
8. Kindly tell me more about ECT/anesthesia guidelines used in your facility?

### SECTION 3: ECT Trained Nurse

1. Describe pre ECT preparation (patient/relative preparation, history, investigations done, examination).
2. Take me through informed consent procedure before ECT is done.
3. What is your role during ECT administration?
4. Who ensures a smooth running of the department?
5. Describe the kind of preparation a patient goes through prior to the attachment of the electrodes.
6. Tell me more about continuous/ maintenance ECT?
7. Describe Post ECT care.
